# Burning mouth syndrome: etiology

**DOI:** 10.1016/S1808-8694(15)30979-4

**Published:** 2015-10-19

**Authors:** Dafne Patrícia Cerchiari, Renata Dutra de Moricz, Fernanda Alves Sanjar, Priscila Bogar Rapoport, Giovana Moretti, Marja Michelin Guerra

**Affiliations:** a Medical Resident in Otorhinolaryngology at the ABC Medical College; b Physician, ENT Specialist, Collaborator in the ABC Medical College; c Intern, 6th year medical student; d Doctor graduated at FMUSP, Full Professor of Otorhinolaryngology at the ABC Medical College; e Medical Resident in Otorhinolaryngology at the ABC Medical College; f Medical Resident in Otorhinolaryngology at the ABC Medical College

**Keywords:** mouth, pain, glossodynia, xerostomy

## Abstract

The Burning Month Syndrome (BMS) is an oral mucosa pain - with or without inflammatory signs - without any specific lesion. It is mostly observed in women aged 40-60 years. This pain feels like a moderate/severe burning, and it occurs more frequently on the tongue, but it may also be felt at the gingiva, lips and jugal mucosa. It may worsen during the day, during stress and fatigue, when the patient speaks too much, or through eating of spicy/hot foods. The burning can be diminished with cold food, work and leisure. The goal of this review article is to consider possible BMS etiologies and join them in 4 groups to be better studied: local, systemic, emotional and idiopathic causes of pain. Knowing the different diagnoses of this syndrome, we can establish a protocol to manage these patients. Within the local pain group, we must investigate dental, allergic and infectious causes. Concerning systemic causes we need to look for connective tissue diseases, endocrine disorders, neurological diseases, nutritional deficits and salivary glands alterations that result in xerostomia. BMS etiology may be of difficult diagnosis, many times showing more than one cause for oral pain. A detailed interview, general physical examination, oral cavity and oropharynx inspection, and lab exams are essential to avoid a try and error treatment for these patients.

## INTRODUCTION

Burning Mouth Syndrome (BMS) is characterized by pain in the mouth with or with no inflammatory signs and no specific lesions. Synonyms found in literature include glossodynia, oral dysesthesia, glossopyrosis, glossalgia, stomatopyrosis, and stomatodynia^1^, ^4^. It usually affects women aged between 40 and 60 years and the prevalence in the general population is 3.7% (1.6% men and 5.5% women)^5^.

BMS generally presents as a triad: mouth pain, alteration in taste, and altered salivation, in the absence of visible mucosal lesions in the mouth^3^.

Pain is a moderate to severe burning sensation, affecting mainly the lateral borders and the tip of the tongue, and may persist for years. Pain may also be present in the gums, the lips and the jugal mucosa, with no visible lesions on oral and pharyngeal examination. Pain increases as the day progresses, in states of anxiety, fatigue, excessive speaking, and when ingesting hot and seasoned food; pain subsides with cold food, work and distraction^3^, ^6^. Burning in the mouth does not match the anatomy of peripheral nerves and typically affects more than one site.

**Chart 1:**
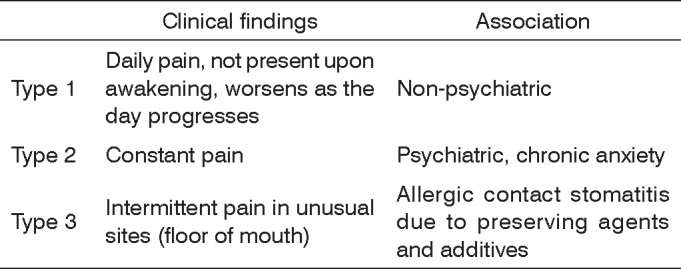
Classification of BMS subtypes^7^

In 1994 Lamey et al.^7^, in an attempt to group different types of patients, divided the syndrome into three types (Chart 1): Type 1 (35%), defined by daily pain where symptoms are absent upon awakening but gradually increase in severity as the day progresses, unrelated to psychiatric conditions. Type 2 (55%) is defined by constant pain day and night; these patients are very anxious. Type 3 (10%) is defined by intermittent pain, with pain free intervals, occurring in non-usual sites such as the floor of mouth and the posterior oropharynx; in this type there is a relation between pain and the type of food taken as well as allergens.

Stinging on the lips and mouth or intense salivation are common symptoms. Alterations in salivary content have been detected such as increased content of potassium, proteins and phosphates, making saliva thicker and stickier. Another important symptom is hypogeusia or dysgeusia, in which patients complain of a persistent metallic, salty or bitter taste^3^.

The etiology of BMS is difficult to define, and there may be more than one etiological factor. Patients seek help from a variety of medical specialists, including dentists, ENT specialists, and dermatologists, and try a range of therapies, the most common of which are: corticosteroids, analgesics, antibiotics, estrogen, retinoids and psychotropic drugs^8^.

A careful clinical history, a general physical examination, a detailed examination of the mouth and oropharynx are mandatory to avoid trial and error treatment of this syndrome.

**Chart 2:**
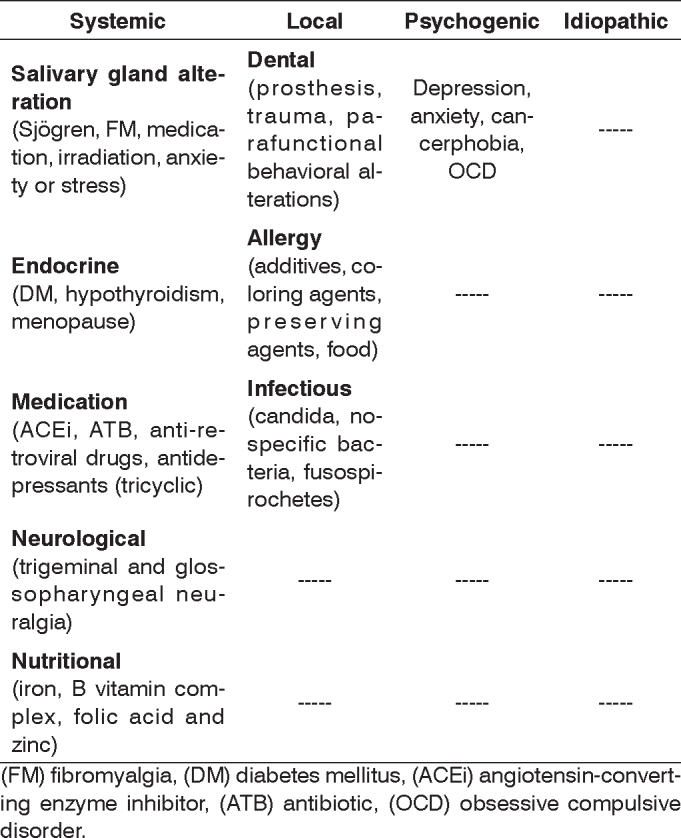
Etiology of BMS

The aim of this review is to provide an overview of possible etiologies of BMS, grouping them into 4 major groups for better understanding (Chart 2): local, systemic, emotional and idiopathic mouth pain. Based on the differential diagnosis of this syndrome, we suggest a protocol to manage these patients.

(FM) fibromyalgia, (DM) diabetes mellitus, (ACEi) angiotensin-converting enzyme inhibitor, (ATB) antibiotic, (OCD) obsessive compulsive disorder.

## REVIEW OF LITERATURE

### A - Local causes

#### Dental

Pain in the mouth may appear due to incorrect use of orthodontic appliances, ill-positioned or corroded dental fixtures, and fixtures used for a very long time, with the patient complaining of a metallic taste. Local reaction caused by contact between the mucosa and metal leads to erythema and a burning sensation. Currently local irritation may also be caused by tongue piercing.

There may be local pain by mechanical trauma in patients that repeatedly bite the jugal mucosa due to dental problems or malocclusion.

Having discarded orofacial pain of dental causes, which is the most common form, parafunctional habits and behaviors should be investigated, such as temporomandibular joint (TMJ)^9^ problems. The main complaints are TMJ crepitation, local pain, and referred pain to the ear or mouth. Patients with miofacial pain, bruxism, large tongues or that press the tongue against the teeth may also have tongue pain.

#### Allergic

Allergy to food coloring agents, preservatives and additives has been identified in 65% of type 3 BMS patients (intermittent pain, with pain free periods, in non-usual sites). Substances identified as commonly causing mouth pain are: cinnamon aldehyde, ascorbic acid, tartarazine, benzoic acid, propyleneglycol and menthol^7^. Food such as shrimp, nuts, fish and chocolate may cause sudden onset allergy, with edema and pruritus usually on the tongue. Severe allergy cases usually are caused by drugs such as sulphonamides, antibiotics, non-steroidal anti-inflammatory medication and analgesics.

Allergy to denture materials is rare, and should be considered only after confirmation by allergy tests which correlate to clinical findings^4^, ^10^.

### Infectious

#### Candida

The causal agent is Candida albicans, and it appears in the elderly, in immunodeficient patients, in prolonged use of antibiotics, immunosuppressant medication, anti-retroviral drugs or corticosteroids. Candida infection may present with or with no inflammation on the mucosa, and patients refer burning in the mouth resulting in dysphagia and sialorrhea^2^. Burning is due to multiplication of candida. Detection of pain stimuli takes place at sensorial nerve terminations called nocireceptors. These small diameter neurons translate mechanical, chemical and temperature variation signals to the central nervous system, giving us the perception of pain or discomfort, acting by means of the receptor capsaicin (vanilloid)^11^.

Dental fixtures also help increase the number of fungal colonies^11^.

#### Non-specific bacteria

This is dental conservation related infection. Causative agents are the polymicrobial oral flora saprophytes including streptococci, enterococci, staphylococci, neisseria, proteus, anaerobes and fusospiral organisms. Many infections are opportunistic. There is gingival congestion and edema of interdental papillae, there may be hyperemia of the mouth mucosa and tongue, which may show indentation marks along its lateral borders. Pain and halitosis are intense in more advanced cases^12^.

### B - Systemic causes

#### Alteration of salivary glands

Salivary gland function is altered by a variety of drugs that induce xerostomia, which affects eating and the capability to resist bacterial colonization of the teeth, and which may alter the taste.

Many drugs may alter salivary gland function, the most common being anticholinergic agents, anti-histamines, anti-retroviral drugs, tricyclic antidepressants, serotonin uptake inhibitors and omeprazol^13^.

Xerostomia depends on the drug, the dose and individual patient differences. Burning may develop days to years after exposure to the causative agent^13^.

Chemotherapy agents, such as adriamycin, cause mucositis 5 to 7 days after the initial dose. The patient presents intense local pain in the oral mucosa, and if chemotherapy is maintained, may develop reduced salivary flow and gland destruction. Normally the gingiva, the dorsal surface of the tongue and the hard palate are not involved^14^.

Radiotherapy (RT) may initially cause mucositis and increased salivary flow due to local inflammation. At this moment the mucosa is extremely painful. If RT is maintained there may be permanent mucosal atrophy, causing burning on the tongue, dry mouth and swallowing difficulty. Salivary gland function is compromised depending on the radiation dose; irradiation over 40Gy/dose causes irreversible gland injury^15^. pH falls significantly from normal values to 6.7514.

#### Medication

There are many description in literature of glossitis in patients taking ACE inhibitors^13^, ^16^, ^17^. Anti-retroviral drugs, antibiotics (cephalosporins, chloramphenicol, penicillin, gabapentin), tricyclic antidepressants and antianxiety drugs are also cited as causing mouth pain^13^. The pain inducing mechanism with no xerostomia is not well known.

#### Connective tissue diseases

Sjögren's syndrome is an autoimmune disease affecting mostly women aged between 40 and 60 years, and may be associated with other connective tissue disorders such as multiple sclerosis and rheumatoid arthritis. The classical presentation of this syndrome is keratoconjunctivitis, xerostomia and connective tissues alterations.

Fibromyalgia (FM) is a chronic pain syndrome usually diagnosed based on the presence of pain in at least 11 of 18 tender points lasting at least 3 months. BMS is seen in 32.8% of FM patients, where the most frequently described oral symptoms include xerostomia (70.9%), orofacial pain (32.8%), TMJ dysfunction (67.6%), dysphagia (37.3%), and dysgeusia (34,2%)^18^.

#### Endocrine

BMS may signal undiagnosed diabetes mellitus (DM), therefore DM should be investigated particularly in patients aged over 50 years, when the incidence of type II DM is increased^11^. Other pain mechanisms in the mouth that should be considered for diabetic patients include candida infection and diabetic neuropathy^19^.

There is controversy in literature on the role of estrogens as an oral mucosa protective factor. Although mouth pain has a high incidence in post-menopausal women, studies have not found a significant relationship between mouth pain and the number of menopausal years, the use of hormone replacement therapy of the number of years of hormone replacement^20^.

#### Neurological

Pain stimuli are detected by nocireceptors, receptors with capsaicin^21^. Petruzzi et al., in a comparative, triple blind study, observed that following capsaicin use during 4 weeks, there was a reduction in mouth pain in 84% of patients, compared to controls. The therapeutic efficacy of capsaicin strengthens the hypothesis that there is a neurogenic cause of BMS.

Further evidence that BMS may have a neurogenic origin is that these patients have thermal hypersensitivity and significant electrophysiological abnormalities compared to controls^22^.

#### Trigeminal neuralgia

Trigeminal neuralgia is characterized by brief episodes of intense pain in bursts, usually in people aged over 50 years, mainly involving the mandibular branch territory, sometimes causing tongue hypoesthesia or paresthesia. Trigeminal neuralgia may be caused by nerve compression due to neoplasms, vascular malformation, encephalopathy, myringomyelia, herpes zoster infection, trauma or after dental extraction^15^.

#### Glossopharyngeal neuralgia

Glossopharyngeal neuralgia is a rare disease occurring mostly in women aged between 40 and 60 years^23^. Pain is usually incapacitating, unilateral, affecting the posterior oropharynx, tonsillar fossa and base of the tongue, extending to the ear. It is initiated by swallowing, coughing or phonation^24^.

Pain may also be caused by undiagnosed neoplasms, infection, elongated styloid process and vascular causes such as arterial compression, elongation or looping, mostly of the posterior inferior cerebellar artery^24^. Neuralgia may course with bradycardia, asystole and syncope in up to 10% of cases^15^.

#### Nutritional deficiencies

A burning sensation on the tongue may occur in 40% of patients with vitamin B deficiency. Tongue pain is usually located on the tip of the tongue, and patients may present papillary atrophy. Patients undergoing hemodyalisis or that restrict their diet in some way (vegetarians, lactose-free diet), alcoholics and the elderly are prone to vitamin B deficiency^15^.

Zinc deficiency may cause organic effects such as lingual papillary atrophy, resulting in dysgeusia and glossodynia. Tanaka et al.^25^ noted improvement in symptoms following zinc intake and further clinical improvement when zinc was associated with B12 vitamin and iron.

#### C - Emotional

Zeller et al.^8^ found up to a 30% psychiatric disorder associated with BMS, including depression, anxiety, obsession, panic syndrome and fear of cancer^2^, ^2^, ^26^ Hakeberg et al.,^1^ focusing psychological aspects of women with BMS, observed that all patients in their study had gone through situations of great stress or disappointment in their lives, culminating with the appearance of mouth pain. These authors also found that these women were very anxious and described themselves as persistent and self-demanding. Vitkov et al.^11^ believe that BMS patients have a reduced pain threshold, and this threshold is lower still in women.

#### D - Idiopathic

This is when careful local, systemic and emotional investigation fails to reveal an etiology for BMS.

## BMS-RELATED ANATOMICAL VARIATIONS

### 1- Lingua plicata

After age four, the tongue may develop grooves in some people, with folds in the anterior two thirds of the tongue. A burning sensation may ensue when there is inflammation along these grooves caused by ingesting acid and/or very seasoned food. These grooves may be entry portals for infection such as herpes simplex, candida and syphilis. This may also be part of Melkerson Rosenthal's syndrome, which progresses with granulomatous glossitis, facial palsy, lingua plicata and lip edema.

It is found in 30% of Down syndrome patients^15^.

### 2 - Geographic tongue

Geographic tongue is characterized by areas of pink mucosa with a grayish center. These areas may converge and are interspaced with normal mucosa. This condition is a benign condition, not a disease. Patients with this lingual alteration may be prone to cancerphobia. Psychological (stress) and local (allergies) reactions are believed to cause this condition, as well as genetic factors. Usually areas of altered mucosa increase when there is some other parallel medical condition^15^. Altered mucosa in this condition is more sensitive to very salty, seasoned or sour food, which may cause pain in the mouth.

## MANAGEMENT OF PATIENTS WITH BMS

BMS is usually multifactorial, and these patients require an organized approach to take the various etiologies into account.

The history of pain should be established, including duration, intensity (on a 1 to 10 scale), site, and factors that improve or worsen the pain. Inquiries should also be made about increased salivation, dry mouth, altered taste, the diet, the use or oral antiseptics, the type of toothpaste, habits such as chewing gum, smoking, and taking alcoholic beverages. Other information includes any relation between pain and the use of dental fixtures and prostheses, parafunctional behavior (bruxism, tongue compression between teeth), and the use of medication and its xerostomia causing potential. A psychological history is also extremely relevant, including questions about anxiety, depression and cancerphobia.

A detailed general and oral physical examination should be made, to uncover the presence of erythema, glossitis, lingual papilla atrophy, sings of tongue biting, candida infection, lingua plicata, geographic tongue, lichen planus or xerostomia.

Work-up should include a complete blood count, TSH, free T4, fasting blood glucose, iron, ferritin, transferrin and folic acid. Diabetics should also be tested for glycosylated hemoglobin to check if diabetes is well controlled. Rheumatologic and autoimmune tests may be required if warranted by the clinical history^3^. Candida cultures are unnecessary as this fungus is part of the normal oral flora; treatment may be given empirically. If food allergy is suspected, patch testing may be needed. Drage et al.^4^ recommends allergic tests in all BMS cases. Electrogustometry may be used to verify salivary flow and content.

Patients with psychological conditions should be referred to a psychiatrist who will assess any relation between the onset of symptoms and events in the patients’ lives such as intense stress, loss of loved relatives, fear of cancer, etc. A dental evaluation should also be done to recommend improved dental care and adequate use of dental fixtures.
